# Ossifying hemangioma of the frontal sinus: a case report

**DOI:** 10.1016/j.bjorl.2023.101341

**Published:** 2023-10-11

**Authors:** Changxing Cao, Shasha Guo, Qiulin Liang, Chao Feng, Maomei Ni, Feng Zhou, Huiping Ye

**Affiliations:** a Guizhou Provincial People's Hospital, Department of Otolaryngology, Guizhou, China; b Guizhou Provincial People's Hospital, Department of Paediatrics, Guizhou, China; c Staff Hospital of Guizhou Province, Department of Pathology, Guizhou, China

## Introduction

Ossifying hemangioma is an exceptionally uncommon condition and has been infrequently documented in clinical practice. In 1974, Natali et al.^1^ were the first to report an ossifying haemangioma of the inferior conchae of the nasal fossae. Moreover, over 50% of cases occur in the vertebrae or skull. Patients typically have no pain or other associated symptoms, and they may only present with a localized skin bulge. These formations are commonly solitary and display a higher incidence in females compared to males. They are predominantly encountered in adults, although individuals of any age can potentially experience their occurrence.^2^ Nonetheless, the causal relationship between osteomas and the development of haemangiomas within the bone, as well as whether haemangiomas stimulate ossification/calcification, remains undetermined. Due to the diversity and reexamination of skull tumors, it is frequently challenging to make differential diagnoses by imaging alone. Hence, the diagnosis is mostly confirmed by biopsy. Accordingly, a case of ossifying hemangioma situated in the frontal bone of the anterior wall of the frontal sinus is presented here for the examination of clinicians.

## Case report

A female patient was admitted to the hospital due to a mass on the left forehead for half a year. Six months ago, the patient noticed a mass on the left forehead, roughly the size of a “rice grain”, with no apparent cause. Intermittently, the mass exhibited slight tenderness and gradual enlargement, while the left eye showed no signs of epiphora, heightened secretion, blurred vision, or diminished visual acuity. Physical examination demonstrated a 2 × 3 cm mass on the left forehead near the upper edge of the eyebrow arch, with a clear boundary, poor mobility, as well as hard texture. Besides, the movement of the left eye in all directions was normal, and no bulge or discharge was observed. Nasopharyngeal fiberoptic laryngoscopy revealed no anomalies in the nasal cavity or pharynx. Following the comprehensive overall assessment and the elimination of surgical contraindications, surgical intervention was carried out under general anesthesia.

**Fig. 1:**
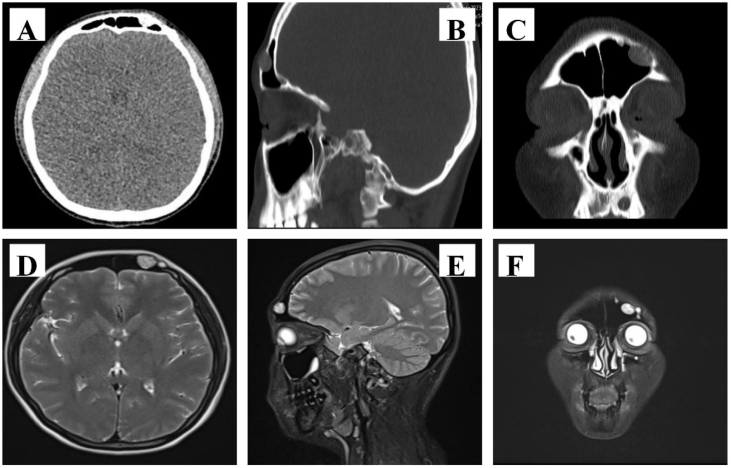
CT of the sinuses showed a frontal sinus mass (A, Position of cross section; B, Sagittal section; C, Coronal section). MRI of the sinuses showed a frontal sinus mass (D, Position of cross section; E, Sagittal section; F, Coronal section).

Sinus Computerized Tomography (CT) demonstrated local bone destruction and soft tissue nodules in the anterior wall of the left frontal sinus. Moreover, the density was uneven, the size was 14 × 10 mm, and the CT value was 130 HU. Furthermore, an MRI of paranasal sinuses plain scan + enhancement: a round equal/slightly long T1 and long T2 nodule with a size of approximately 11 × 14 mm were found in the left part of the frontal bone, which grew into the frontal sinus, with a clear boundary. Following the enhanced scan, it was a moderately heterogeneous enhancement, and no conspicuous swelling of the surrounding soft tissue of the scalp was observed (Fig. 1).

**Fig. 2:**
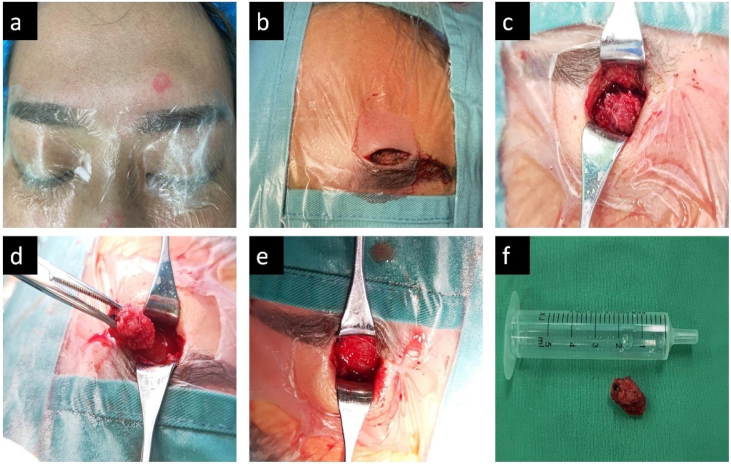
Preoperative localization of the raised mass on the upper edge of the left eyebrow arch (a). The incision was designed at the eyebrow arch (b). The mass was exposed while the surrounding area of the mass was sanded with a skull rotation (c). The bone mass in the frontal sinus was removed (d). After the bone mass of frontal sinus was completely removed, the mucosa of frontal sinus was well preserved without damage (e). The whole frontal sinus bone mass was taken out for measurement and comparison (f).

For aesthetic consideration, a transverse skin incision measuring 2 cm in length was created in the vicinity of the left eyebrow arch, adjacent to the mass. The skin, subcutaneous tissue, and muscle were separated to expose the frontal bone mass. Furthermore, the surface membrane of the frontal bone mass was detached using an orthopedic peeling instrument, revealing both the mass and the neighboring healthy bone in their entirety. Subsequently, the bone mass was fully isolated. Examination illustrated that the mucous membrane of the frontal sinus cavity was intact, the sinus cavity was not broken, and no remnants of the bone mass were evident. Following the operation, the group layer was pressurized and sutured, and the skin was sutured (Fig. 2).

**Fig. 3:**
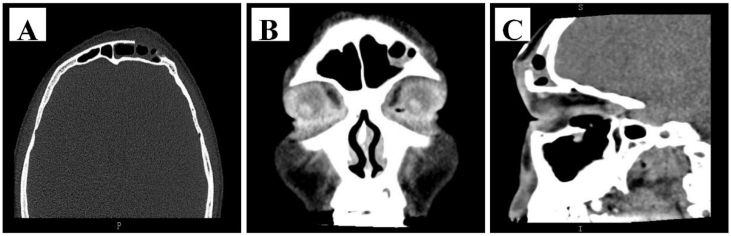
Postoperative sinus CT was performed: (A) Position of cross section; (B) Coronal section; (C) Sagittal section.

Histopathological examination indicated an ossifying hemangioma with a small amount of surrounding bone tissue, and microscopically, mature trabecular bone, as well as multiple small vascular Spaces, were demonstrated (Fig. 3 at various multiples). Moreover, the final diagnosis was ossifying hemangioma of the frontal sinus.

**Fig. 4:**
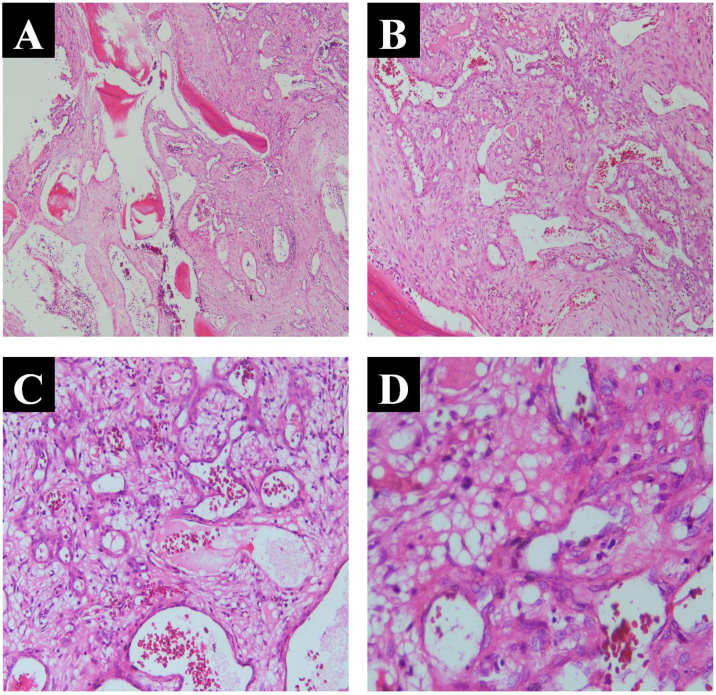
Ossifying hemangioma of the frontal sinus showed mature trabecular bone and multiple small vascular Spaces under different microscope magnification (H&E, A, Enhanced 40×; B, Enhanced 100×; C, Enhanced 200×; D, Enhanced 400×).

Following a short-term follow-up, a sinus CT reexamination of the patient was performed, which further indicated that the irregular bone mass in the frontal sinus disappeared without residue, skull discontinuity, bone defect in the surgical area, and an opaque shadow characterized by soft tissue density was detected within the sinus cavity. The patient’s condition was attributed to bleeding, with no indications of frontal emphysema or hematoma observed. The patient remained in a state of clinical remission (Fig. 4).

## Discussion

X-Ray findings of intraosseous hemangioma of the skull indicate honeycomb-like, extensive, well-defined sparse areas in axial view and a classic sunray pattern of trabeculation on tangential views. There is typically no reactive sclerosis at the margins.^3^ Certain instances manifested solely as a lytic or densely expandable bone mass. Frontal bone hemangiomas commonly exhibit outward expansile growth, with intracranial growth being a rare occurrence.

CT revealed an intradiploic lytic mass with rarefaction and a honeycomb pattern. Moreover, CT is more beneficial than Magnetic Resonance Imaging (MRI) imaging or other neuroimaging modalities in planning surgery due to the fact that it indicates the site and extent of the tumor better in bone windows. TI and T2-weighted MR Imaging showed long signals, and the lesions showed different degrees of heterogeneous enhancement. Furthermore, the display of this imaging reflects the low-fat content of ossified hemangiomas accompanied by varying degrees of blood flow signals. Hence, the slightly prolonged T1 and T2 signals observed in this particular case are indicative of the lesion's comparatively diminished fat content. The high signal intensity on T2-weighted MRI may be induced by blood pooling or slow flow, and enhanced MRI scans suggest moderately heterogeneous enhancement.

The ossifying haemangioma of the frontal sinus did not exhibit any indications of deleterious attributes and phenotypically appeared as an osteoma. It is frequently identified at an early stage by the appearance of raised features on the surface. Nonetheless, numerous types of intraosseous tumors grow slowly. At times, in the early stages, it may manifest without discernible clinical symptoms or signs, which is challenging to diagnose. Consequently, they are typically observed conservatively until the late stage, when relevant symptoms and signs appear and are eventually confirmed by surgical resection and pathological examination.^4^ In our case, we surgically removed the abnormal tissue without encountering any complications during the postoperative period, and there was no significant occurrence of excessive blood loss. Owing to no critical surgical complications occurring, no additional changes to the surgical procedure were necessary.^5^

For ossifying hemangioma of the frontal bone, complete resection is widely regarded as a benign tumor, and recurrence can be avoided. Follow-up CT scans should be performed 6–12 months after surgery to assess recurrence, to detect late adverse reactions related to treatment. In summary, based on the information available to us, this represents a comprehensive account of a characteristic and exceedingly uncommon instance of ossifying hemangioma within the frontal sinus.

## Conclusion

Ossifying hemangioma of the frontal sinus is extremely rare in clinical practice. It grows slowly and has no clinical-related symptoms in the early stage. The size, location, and surrounding tissue of the tumor can be preliminarily judged by imaging examination. Surgical excision stands as the singular efficacious approach for addressing ossifying hemangioma within the frontal sinus, with no instances of reoccurrence after the surgical intervention. Both otolaryngologists and neurosurgeons should maintain a heightened vigilance regarding the clinical indicators and manifestations of this condition, thereby facilitating informed and prudent decision-making.

## Ethical approval

All procedures performed in studies involving human participants were in accordance with the ethical standards of the institutional and national research committee and with the 1964 Helsinki declaration and its later amendments or comparable ethical standards. Book consent has been obtained from the patient for publication of all relevant data.

## Funding

The authors received no financial support for this article’s research, authorship, or publication.

## Conflicts of interest

The authors declare no conflicts of interest.
